# Novel metabolic adaptation driven by glycoside hydrolase family 25 protein contributes to increasing trimethoprim-sulfamethoxazole resistance in clinical human *Brucella melitensis* isolates in China

**DOI:** 10.1128/aac.01284-25

**Published:** 2026-01-22

**Authors:** Xiaowen Yang, Wenqing Ning, Yaqin Yuan, Xuexin Hou, Shengxin Ge, Hai Jiang, Xiaowei Peng, Tianqi Xue, Hongyan Zhao, Biao Kan, Jiabo Ding

**Affiliations:** 1 Key Laboratory of Animal Biosafety Risk Prevention and Control (North), Ministry of Agriculture and Rural Affairs, Institute of Animal Science, Chinese Academy of Agricultural Sciences 12661, Beijing, People's Republic of China; 2 National Key Laboratory of Intelligent Tracking and Forecasting for Infectious Diseases, National Institute for Communicable Disease Control and Prevention, Chinese Center for Disease Control and Prevention 96698, Beijing, People's Republic of China; 3 College of Veterinary Medicine, Shandong Agricultural University 34734, Tai'an, Shandong, People's Republic of China; 4 Key Laboratory of Jiangsu Preventive Veterinary Medicine, Key Laboratory of Avian Preventive Medicine, Ministry of Education, College of Veterinary Medicine, Yangzhou University 38043, Yangzhou, Jiangsu, People's Republic of China; 5 National Reference Laboratory for Animal Brucellosis, China Institute of Veterinary Drug Control 620909, Beijing, People's Republic of China; Columbia University Irving Medical Center, New York, New York, USA

**Keywords:** *Brucella*, trimethoprim-sulfamethoxazole resistance, wgSNPs, glycoside hydrolase family 25 protein, metabolic adaptations

## Abstract

Brucellosis, caused by *Brucella* spp., is a globally zoonotic disease that results in substantial economic losses and public health concerns. Although antibiotic-resistant *Brucella* strains have been reported worldwide, the current status and underlying mechanisms of resistance among Chinese isolates remain poorly characterized. In this study, we analyzed 636 clinical human isolates of *B. melitensis* from China using genomic sequencing, transcriptomic sequencing, and neural network prediction to identify key determinants and mechanisms of antibiotic resistance. Functional validations were performed using gene editing and protein-protein interaction assays. We found a gradual increase in resistance to trimethoprim-sulfamethoxazole (SXT) among Chinese isolates in recent years, despite the absence of known antibiotic resistance genes. Comparative genomic analyses between high- and low-minimum inhibitory concentration (MIC) isolates revealed specific single nucleotide polymorphisms (SNPs) that were present only in high-MIC isolates. Transcriptomic analysis demonstrated that high-MIC and low-MIC isolates activated distinct metabolic pathways in response to SXT exposure. Notably, genes influenced by specific SNPs exhibited opposing expression patterns after SXT treatment. Gene-editing experiments revealed that deletion of the glycoside hydrolase family 25 (GH25) gene, which was identified through SNP analysis, was associated with SXT resistance and notably altered *Brucella* energy metabolism, although it did not impact virulence in host cells. Further, we identified a direct interaction between GH25 and XylF. Collectively, our study reveals a novel genetic mechanism driving SXT resistance in *B. melitensis*. These findings highlight the critical need for vigilant surveillance of antibiotic resistance to mitigate public health risks associated with the potential widespread emergence of antibiotic resistance.

## INTRODUCTION

Brucellosis has a significant impact on public health and economic development. It is a zoonotic disease caused by gram-negative facultative intracellular bacteria, *Brucella* spp. Humans usually acquire infection through direct contact with infected animals or by consuming inadequately sterilized products from infected animals (1). The disease causes large annual economic losses, mainly due to loss of labor in humans and reproductive disorders in animals (2). Recent global data indicate a resurgence of both human and animal brucellosis, with approximately 2.1 million new human brucellosis cases reported each year (3). This number is much higher than the early WHO estimate of ~500,000 cases per year (4), which likely underestimated the true burden. Rapid economic development and the increased trade and transportation of livestock and animal products have facilitated the spread of *Brucella* spp. (5). The pathogen infects a wide range of hosts, including domestic livestock, wildlife, and even marine mammals, which makes prevention and control extremely challenging at the global level.

Brucellosis is particularly severe in China (6). It is the fastest-growing zoonotic disease among notifiable infectious diseases (https://www.ndcpa.gov.cn/jbkzzx/c100016/common/list.html) in recent years. Trimethoprim-sulfamethoxazole (SXT) is recommended globally, including in China, as an effective treatment for brucellosis in pregnant women and children under 8 years of age (7–9). SXT is a fixed-ratio combination of trimethoprim (TMP) and sulfamethoxazole (SMZ). It inhibits bacterial nucleic acid synthesis through dual metabolic blockade and thus exerts a bacteriostatic effect (10). However, SXT-resistant *Brucella* strains have been increasingly reported worldwide (11). In China, several recent studies have also reported SXT-reduced-susceptibility or SXT-resistant *Brucella* isolates (12, 13). The emergence and spread of such strains make clinical management and public health control more difficult. International travel, the movement of infected livestock, and contaminated animal products can all accelerate dissemination.

Previous studies have linked SXT resistance in *Brucella* spp. mainly to the *folA* and *folP* genes, in which deletions or mutations confer resistance (14). Yet, for Chinese isolates, the resistance genes and the detailed mechanisms have not been well studied. Therefore, the aims of this study were: (i) to determine the minimum inhibitory concentration (MIC) of SXT for 636 clinical isolates of *B. melitensis* collected from different regions of China between 2010 and 2021, and to analyze temporal and geographical trends of SXT resistance; (ii) to identify novel genes associated with SXT resistance through neural network prediction, comprehensive genomic and transcriptomic analyses; and (iii) to clarify the novel molecular mechanisms responsible for SXT resistance in *B. melitensis*. Our findings provide new insights into SXT resistance mechanisms and supply a basis for developing better therapeutic and preventive strategies for brucellosis in China and globally.

## MATERIALS AND METHODS

### Relevant sequence acquisition and culture of *Brucella* strains

The genome, amino acid, and nucleic acid sequences of *B. melitensis* bv. 1 16M, *B. melitensis* bv. 2 63/9 (used as the reference genome for alignment and single nucleotide polymorphism [SNP] calling), *B. melitensis* bv. 3 ether, and *B. melitensis* M5 (used as the parental strain for constructing gene-deletion and complementation strains) were downloaded from the NCBI RefSeq database (https://www.ncbi.nlm.nih.gov/datasets/genome/?taxon=29459). All cultures and inactivated samples of reference strain and clinical isolates were prepared in a BSL-3 laboratory of the National Institute for Communicable Disease Control and Prevention of the Chinese Center for Disease Control and Prevention. Other experiments, including SXT MIC determination and total RNA extraction, were also performed in the BSL-3 laboratory.

### MIC value detection for SXT

Following a modified protocol from reference 15, SXT (TMP and SMZ in a 1:19 ratio) was diluted to final concentrations of 0.06/1.2 to 32/608 µg/mL. Then, 100 µL diluted solution with Brucella broth (BD, USA) media (approximately 1 × 10^5^ CFU/well) was added to 96-well plates. MIC values were recorded after 48 h of incubation. MICs were also read every 24 h to plot time-MIC curves. Reference strains were used as positive controls. Drug-free medium was used as the negative control. *Escherichia coli* ATCC 25922 served as the quality control strain.

### DNA extraction, genomic sequencing, and analysis

Strains were cultured to an OD_600_ of 0.6–0.8. DNA was extracted using the Wizard Genomic DNA Purification Kit (Promega, USA) according to the manufacturer’s instructions. DNA concentration was measured using a Qubit 4 Fluorometer. Then, 100 ng of DNA was used for library preparation and sequencing. Genomic DNA was fragmented, end-repaired, adaptor-ligated, and PCR-amplified using the MGIEasy Fast PCR-FREE Kit (BGI, China). The double-stranded PCR products were heat-denatured and circularized with a splint oligo. The single-stranded circular DNA (ssCir DNA) was used as the final library. DNA nanoballs (DNBs) containing >300 copies were generated by rolling-circle replication. The libraries were sequenced on the MGISEQ-2000 platform (BGI) using the MGISEQ-2000RS high-throughput kit (PE150) (BGI). The sequencing depth was >200×. Raw reads were filtered to obtain clean data. SNPs and InDels were identified using pipelines reported in our previous studies (15, 16). High-impact SNPs and InDels were further validated by amplifying and Sanger sequencing the 300 bp flanking regions. Validated SNPs and InDels present in isolates but absent in reference strains were analyzed further.

### RNA extraction and transcriptome analysis

Total RNA from isolates and reference strains was extracted using previously established methods (17). RNAprotect Bacteria Reagent (Qiagen, Germany) was added before extraction. DNase I (TaKaRa Bio, Japan) was used to remove DNA. RNA concentration and quality were measured using a NanoDrop and an Agilent 2100 bioanalyzer. rRNA was removed using the Ribo-Zero Magnetic Kit (EpiCentre, USA). RNA-seq libraries were prepared with the TruSeq Stranded Total RNA Library Prep Kit (Illumina, USA). RNA fragments were reverse-transcribed, converted to double-stranded cDNA, adaptor-ligated, purified, and quantified. Sequencing was performed on the Illumina HiSeq 4000 platform (Illumina), and data were analyzed on the Majorbio online analysis platform (Majorbio, China). Differentially expressed genes were identified using an adjusted *P* value ≤ 0.05 and absolute value of log2 ratio ≥ 2.

cDNAs were synthesized using the PrimeScript RT Reagent Kit (TaKaR). PCR was performed with the primers shown in Table S1 to evaluate the Ct (cycle threshold) value. Relative quantitative method (2^−ΔΔC*t*^) was used to compare gene expression levels, normalized to the 16S rRNA expression level in the reference strain.

### Neural network prediction of SXT resistance genes

Following reference (18), various molecular fingerprints of TMP and SMZ were computed. Using the 2D Tanimoto coefficient, similar compounds and their target information were matched from the ChEMBL database (https://www.ebi.ac.uk/chembl/). Protein sequences of *Brucella* spp. were downloaded from UniProt (https://www.uniprot.org/uniprotkb?query=Brucella). After removing redundant and low-quality sequences, these proteins were used as potential targets. Deep learning algorithms and neural networks predicted interactions between TMP/SMZ and *Brucella* proteins. Based on the number of predicted targets and on already reported SXT targets in *Brucella*, the model parameters were adjusted several times. The top 20 proteins ranked by integrated scores were selected. Identified protein types were cross-referenced with proteins in the *B. melitensis* bv. 1 16M genome to select candidate target proteins. The algorithm and model were provided by APExBIO (Houston, USA).

### Gene deletion and complementation

Gene deletion and complementation strains were constructed as previously described (17). The homologous-recombination plasmid pgex-18AP was used for gene deletion. Primers for gene deletion and complementation strains are listed in Table S1. PCR products were cloned using ClonExpress MultiS One Step Cloning Kit (Vazyme, China) and transformed into DH5α competent cells (CWbio, China). Positive plasmids were electroporated into *Brucella* strains. Positive colonies were selected by double screening based on the plasmid-borne resistance marker and the *secB* counterselection gene to obtain the deletion strains. Complement strains were constructed using the pBBR1MCS plasmid. Target gene sequences with promoter regions were amplified (Table S1). Complement strains with SNPs were constructed by target sequence from isolates with the same methods.

### Growth characteristics *in vitro*

For growth analysis under SXT (0.24/4.8 µg/mL) *in vitro*, one colony of *Brucella* strain was inoculated into 3 mL of Brucella broth medium and cultured for 24 h at 37°C in a shaking incubator at 200 rpm. Cultures were adjusted to the same concentration (OD_600_ ≈ 0.4) for growth curve analysis. A 50 μL sample was inoculated into 5 mL medium with/without SXT. The cultures were incubated at 37°C with shaking at 200 rpm, and the OD_600_ value was determined at different time points. Strains were tested in triplicate in three independent experiments.

### Biochemical parameter assay

Cultures with an OD_600_ of 0.4–0.6 were centrifuged to collect the cells, which were then washed three times with PBS. The cells were resuspended in PBS supplemented with SXT and incubated for 2 h before cell collection for subsequent experiments.

#### NAD^+^/NADH determination

Total NAD^+^ and NADH levels were measured following the manufacturer’s instructions for an NAD^+^/NADH Assay Kit with WST-8 (Beyotime, China). The absorbance was detected at 450 nm. The NAD^+^/NADH ratio was calculated using a standard curve established with the standard products to determine the intracellular NAD^+^ and NADH levels.

#### LDH release assay

The supernatants were collected, and the LDH Cytotoxicity Assay Kit (Beyotime) was followed. The samples were incubated at room temperature in the dark for 30–60 min before the absorbance was measured at 490 nm. Maximum release wells and blank control wells were used to calculate the cell death rates.

#### Total ROS (reactive oxygen species) measurement

With minor modifications to the method described (19), a Reactive Oxygen Species Assay Kit (Beyotime) was used. After treatment with different concentrations of SXT, the cultures were incubated in the dark for 2 h at 37°C with shaking at 220 rpm. Fluorescence was measured at an excitation wavelength of 488 nm and an emission wavelength of 525 nm using a microplate reader. Each SXT concentration was tested in three independent experiments.

### Eukaryotic protein expression *in vitro*, pull-down, and LC-MS/MS analysis

Primers (Table S1) were designed to clone genes into the pCMV-1 (Myc-tag) and pCMV-7 (3×Flag tag) plasmids. Well-growing HEK 293T cells were spread into 6-well plates for protein expression. Correctly sequenced plasmids were transfected into the HEK 293T cells using jetPRIME transfection reagent (Polyplus, France) following the manufacturer’s instructions. The cells were cultured for 24–48 h before sample collection. The cells were centrifuged at 1,000 rpm for 5 min, lysed with precooled lysis buffer containing inhibitors, and gently vortexed to ensure thorough lysis. Protein expression was detected via Western blot (WB) analysis with corresponding antibodies (Beyotime).

A Myc-tag Protein IP Assay Kit with Magnetic Beads (Beyotime) was used for pull-down assay following the manufacturer’s instructions. Proteins with Myc-tag were bound to magnetic beads. *Brucella* cultures were collected by centrifugation, quick-frozen in liquid nitrogen for 15 min, and lysed two times. Lysates were incubated on ice for 4 h, added to columns with bound protein, and incubated with shaking at 4°C for 4 h before washing several times and eluting interacting proteins. Eluates from columns with only carrier plasmid protein and *Brucella* proteins served as experimental controls.

Nano-LC-MS/MS analysis was performed using an Orbitrap Fusion Tribrid MS (Thermo, USA) instrument equipped with a nanospray flex ion source coupled with a Dionex UltiMate 3000 RSLC nanosystem (Thermo, USA). The raw data files were searched against the *B. melitensis* proteome from the UniProt database using MaxQuant. The proteins and peptides were filtered with a false discovery rate (FDR) <1%. The enzyme parameters were limited to semitryptic peptides with a maximum miscleavage of 2.

### Co-immunoprecipitation (Co-IP) assay

Co-IP assays were performed using a Flag-tag Protein IP Assay Kit with Magnetic Beads (Beyotime). Approximately 2.5 μg of each plasmid was transfected into HEK 293T cells, and the cells were collected after 24–48 h of culture. The collected cells were lysed on ice. Part of the supernatant was denatured as input samples, and the rest was incubated with the prepared magnetic bead suspension overnight at 4°C. After incubation, the beads were washed and the eluates were denatured as IP samples. WB was performed using the corresponding primary and secondary antibodies, with GAPDH serving as the internal control protein.

### Confocal laser scanning microscopy analysis

Well-grown HeLa cells were spread onto eight-well chamber slides (Beyotime), and the plasmids were transfected. Cells were cultured for another 24 h before the old medium was discarded. The cells were gently rinsed with PBS, fixed with 4% PFA, permeabilized with 0.1% Triton X-100 in 5% BSA, and blocked with 5% BSA. Primary and secondary antibodies diluted in PBS were added and incubated at room temperature. The nuclei were stained with DAPI (Beyotime). The samples were observed under a confocal laser scanning microscope (Leica, Germany).

### Cell infection assay

Following reference 20 with modifications, cell infection assays were performed. The suspension was adjusted to approximately 0.5 McF. RAW264.7 cells were cultured, resuspended in DMEM with 1% FBS, and counted using a cell counter (Invitrogen, USA). Approximately 2.5 × 10^5^ cells per well were seeded into 24-well plates and cultured for 12 h before the infection assay. The suspension was diluted to an MOI of 1:100 to 1:200 and added to each well. After centrifugation at 1,000 rpm for 10 min, the plates were incubated at 37°C with 5% CO_2_ for 1 h. The medium was then replaced with DMEM containing 1% FBS and 25 μg/mL gentamicin. Cells were collected at various time points to determine the intracellular load.

### Data analysis and visualization

Statistical analyses were performed using Excel 2010 and SPSS 23.0. A *P* value < 0.05 was considered significant for one-way analysis of variance (ANOVA). The phylogenetic tree was drawn by iTOL (https://itol.embl.de/). Other figures were created using GraphPad Prism 5.

## RESULTS

### Emerging SXT resistance in *B. melitensis* isolated from China

**Fig 1:**
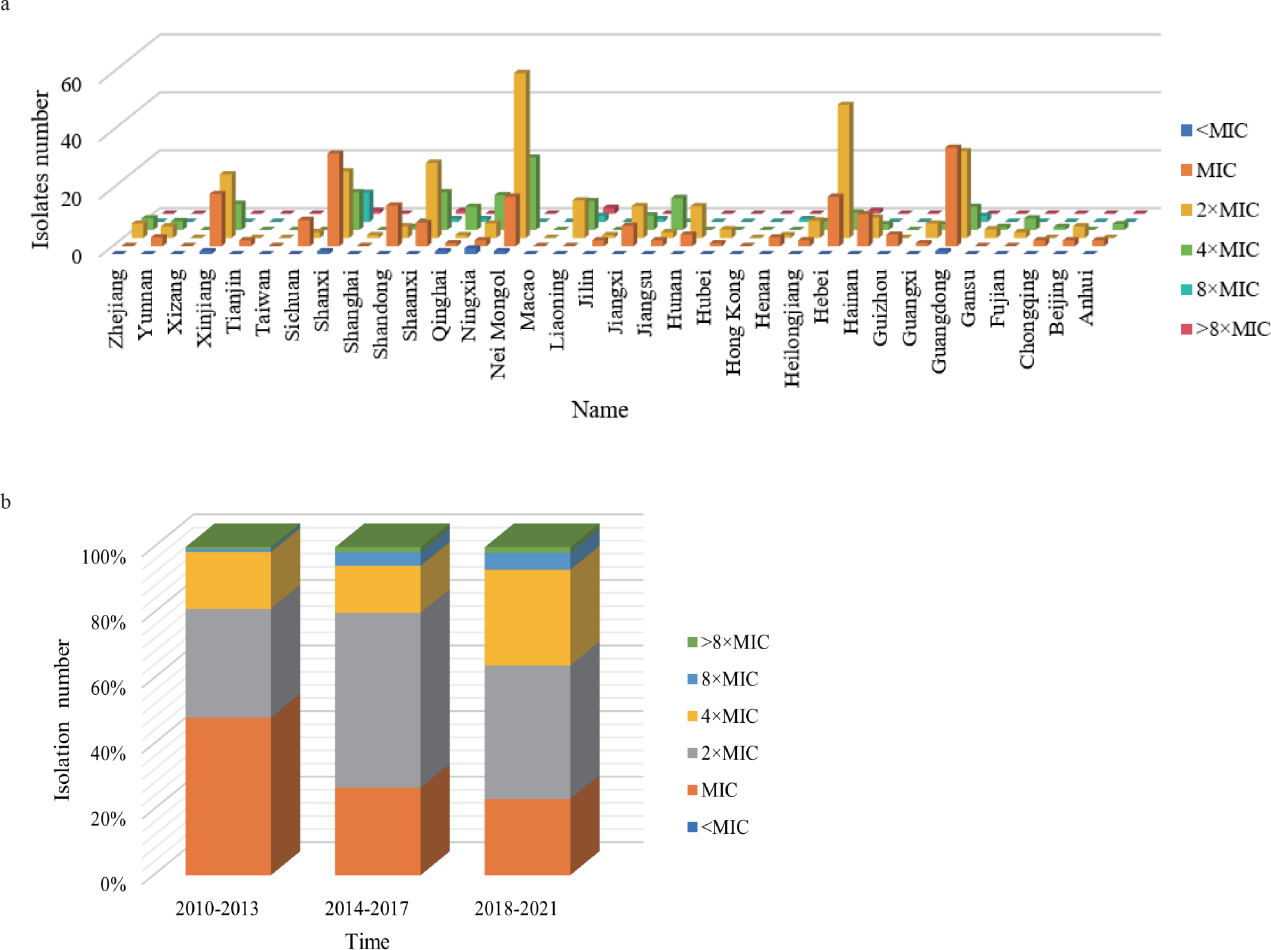
Geographic distribution and MIC values of *B. melitensis* isolates in China. (**a**) Regional distribution and MIC values of isolates. (**b**) Proportion of isolates with different MIC values over time, highlighting the increasing prevalence of high-MIC isolates.

This study evaluated the SXT MICs of 636 human clinical *B. melitensis* isolates collected from different regions of China between 2010 and 2021. Isolates from regions with high brucellosis endemicity (Xinjiang, Inner Mongolia, Shaanxi, Shanxi, and Ningxia) tended to have higher SXT MICs than isolates from regions with low or sporadic brucellosis (Jiangsu, Hainan, Guangxi, and Shanghai). The results indicated that *B. melitensis* isolates from brucellosis-endemic regions in China showed lower susceptibility to SXT (Fig. 1a). MIC values of *B. melitensis* bv. 1 16M, *B. melitensis* bv. 2 63/9, and *B. melitensis* bv. 3 ether were 0.03/0.6 μg/mL (TMP/SMZ), 0.03/0.6 μg/mL, and 0.06/1.2 μg/mL, respectively. In this study, 0.06/1.2 μg/mL was used as the baseline MIC for analysis. The MIC values for Chinese isolates ranged from 0.03/0.6 to 2/38 μg/mL (Fig. 1a). Approximately 24.21% of isolates had MICs above the baseline. Northern provinces showed more high-MIC isolates than southern provinces. Based on CLSI recommendations (broth dilution method, CLSI M45) (21), an MIC of SMZ/TMP greater than 2/38 μg/mL indicates resistance. Five isolates met this criterion (4/96 μg/mL at 72 h). These five isolates came from Liaoning, Ningxia, Hebei, Shaanxi, and Shanxi.

When isolates were analyzed in 4-year intervals, this study observed a steady increase in MICs. Since 2010, the proportion of isolates at baseline MIC decreased over time. In contrast, isolates with 4× MICs and 8× MICs increased, indicating an emerging SXT-resistance trend (Fig. 1b). This trend was most obvious in Xinjiang Uygur Autonomous Region (referred to as Xinjiang), where isolates collected after 2018 mainly showed 4× MICs (3/9, 33.33%) and no baseline MICs were observed.

### Specific SNPs associated with SXT resistance in Chinese isolates

Previous studies linked *folA* and *folP* genes to SXT resistance in *Brucella* spp. (22), while other bacteria show resistance via genes such as *sul* and *drfA* (23). Additionally, our previous studies indicated that *Brucella* could acquire resistance genes via transposition (24). In this study, PCR screening of 174 isolates with 4× MICs did not detect deletions in *folA* or *folP* and did not detect acquisitions of *sul* or *drfA*. This suggested that Chinese isolates might use alternative SXT-resistance mechanisms.

**Fig 2:**
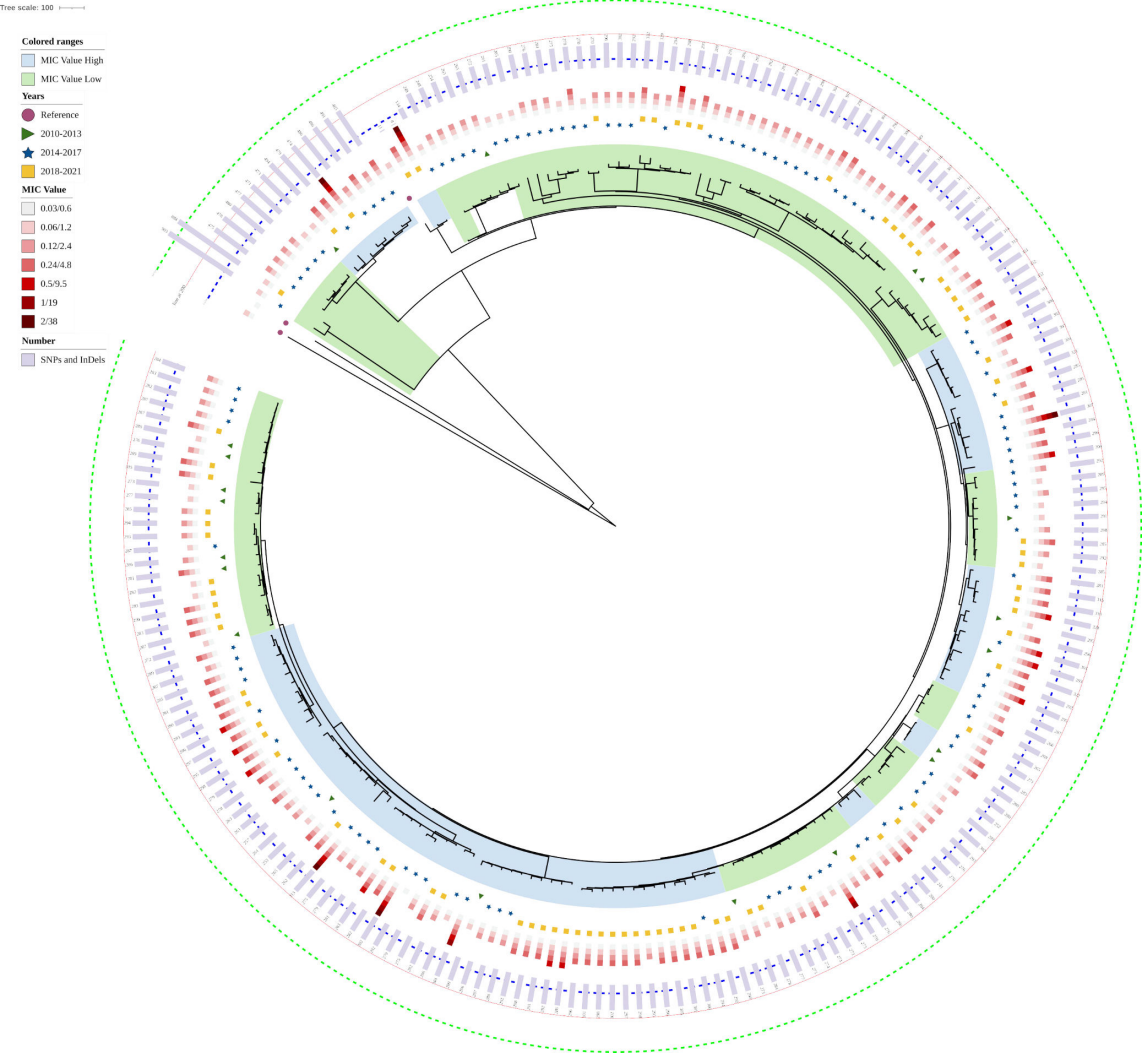
Genetic evolution analysis of *B. melitensis* isolates in China. The phylogenetic tree constructed using whole-genome SNPs (wgSNPs) across isolates from various regions and time points. Scale represents 100 SNPs. Dashed green, red, and blue lines indicate boundaries of 800, 200, and 100 SNP differences, respectively, compared to the reference strain.

Whole-genome sequencing of 212 isolates (192 with MICs >2× MIC and 20 baseline MIC isolates) was performed. The isolates showed a high level of relatedness and were close to the reference strain *B. melitensis* 63/9 (Fig. 2). Compared with the reference strain, 13 isolates had >800 SNPs, and 2 isolates had <100 SNPs. More than 80% of isolates had 100–400 SNPs. The phylogenetic tree showed that isolates with the same MIC level did not cluster into an independent branch. There was also no obvious separation between the >2× MIC group and the baseline MIC group.

We therefore screened for SNPs and InDels that were shared by the >2× MIC isolates, and we removed the SNPs and InDels that already existed in the three *B. melitensis* reference strains (biovars 1–3). After this filtering, we identified 179 common SNPs/InDels. These variants were annotated. SNP and InDel analyses revealed 175 affected genes in high-MIC value isolates (Fig. S1). After excluding genes with low variation impact (intergenic, upstream/downstream, and synonymous variations), 79 genes were potentially associated with higher MIC values (Table S2). Among these, eight genes carried frameshift mutations. These 179 specific SNPs therefore likely contribute to the increased SXT MICs observed in Chinese isolates.

### Metabolic adaptations in Chinese SXT-resistant isolates

**Fig 3:**
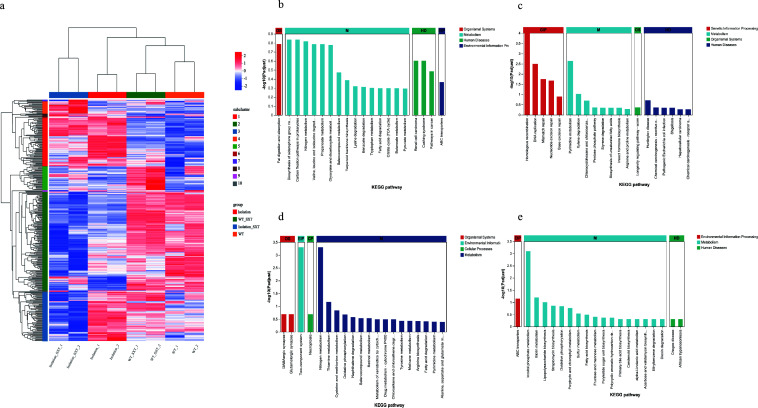
Distinct metabolic pathways activated by the reference strain and resistant isolates in response to SXT. (**a**) Heatmap of differential gene expression in reference and resistant isolates cultured in Brucella broth with and without SXT (0.24/4.8 μg/mL). WT and WT_SXT denote reference strain without and with SXT treatment; Isolation and Isolation_SXT denote resistant isolate without and with SXT treatment, respectively. KEGG functional clustering of significantly upregulated genes in (**b**) reference strain and (**c**) resistant isolate, and significantly downregulated genes in (**d**) reference strain and (**e**) resistant isolate, after SXT treatment.

Previously, we established methodologies combining transcriptomics and neural network predictions to identify novel resistance genes (17, 19). Here, transcriptomic analysis comparing *B. melitensis* bv.3 ether (reference strain) and a high-MIC clinic isolate revealed distinct gene-expression patterns and signaling pathways (Fig. 3a; Table S3). After SXT exposure, the reference strain mainly upregulated genes involved in siderophore nonribosomal peptide biosynthesis and downregulated nitrogen metabolism genes (Fig. 3b and c). In contrast, the resistant isolates prominently upregulated genes associated with homologous recombination, pyrimidine metabolism, DNA replication, and mismatch repair, and downregulated genes involved in inositol phosphate and biotin metabolism (Fig. 3d and e).

Neural network analysis validated the known resistance gene *folA* (DK62_RS00200), indicating that the prediction pipeline was reliable. Then this study annotated the top 20 predicted genes. Many of them did not have clear KEGG assignments, but two genes were mapped to the nitrogen-metabolism pathway (Table S4). Expression analysis further showed that 18 genes displayed opposite expression patterns between the reference and the resistant strains after SXT treatment. These genes may play critical roles in SXT resistance.

### Glycoside hydrolase family 25 (GH25) protein mediates SXT resistance

Genomic analyses have linked specific SNPs to bacterial antibiotic resistance (25). We focused on SNPs causing frameshift mutations, because such mutations usually have a strong functional impact. Among the eight genes with frameshift mutations, seven were upregulated in the reference strain after SXT exposure, whereas five were downregulated in the resistant strain. Crucially, *ccmB*, *mltA*, *aroE*, and GH25 exhibited divergent expression patterns between strains.

**Fig 4:**
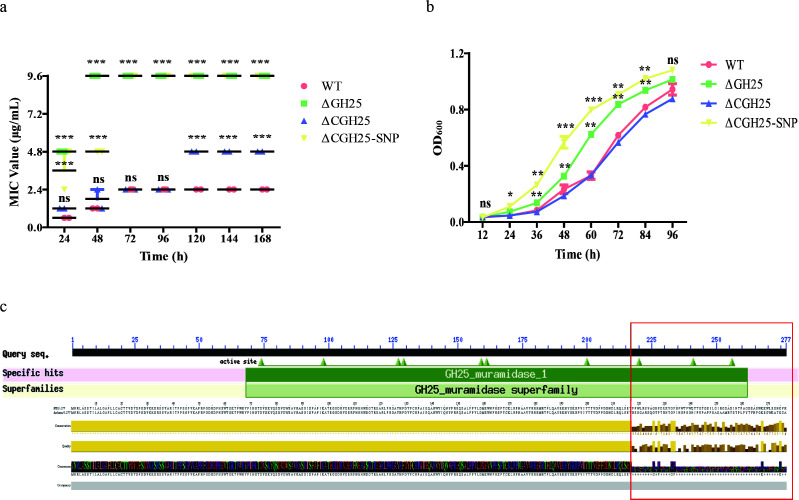
GH25 involvement in SXT resistance in *B. melitensis*. (**a**) MIC values for SXT measured *in vitro* for WT, ΔGH25 (deletion strain), ΔCGH25 (complementation strain), and ΔCGH25-SNP (mutant complementation strain). (**b**) Growth curves of strains cultured with SXT (0.24/4.8 μg/mL). (**c**) Conserved domain analysis of GH25 protein, highlighting the specific SNP site (183Adel) and subsequent significant amino acid changes post-mutation. Significance levels: *P < 0.05, **P < 0.01, ***P < 0.001, and ns, not significant.

Among these discordantly expressed genes deletion strains constructed in this study, we found that GH25 deletion significantly increased MICs, while complementation with a wild-type (WT) GH25 gene reversed this effect (Fig. 4a). In SXT-containing medium, the GH25 deletion and the SNP-complemented strains grew faster than the WT and complement strains (Fig. 4b). Genomic sequencing revealed an SNP-induced frameshift mutation in GH25 (183A deletion). This mutation resulted in a truncated GH25 protein, with the truncation occurring beyond amino acid 218 (Fig. 4c). Notably, this SNP was detected in all 179 isolates with MIC values >2× MIC, suggesting that this SNP is widespread among Chinese SXT-non-susceptible *B. melitensis* isolates. qPCR analysis confirmed drastically reduced GH25 expression in resistant strains (Fig. S2), aligning with transcriptomic data (Table S3). Collectively, these results indicate that mutations in GH25 contribute to high-MIC values in clinical isolates.

### GH25 influences energy metabolism through interaction with sugar transport ABC transporter substrate-binding protein XylF

**Fig 5:**
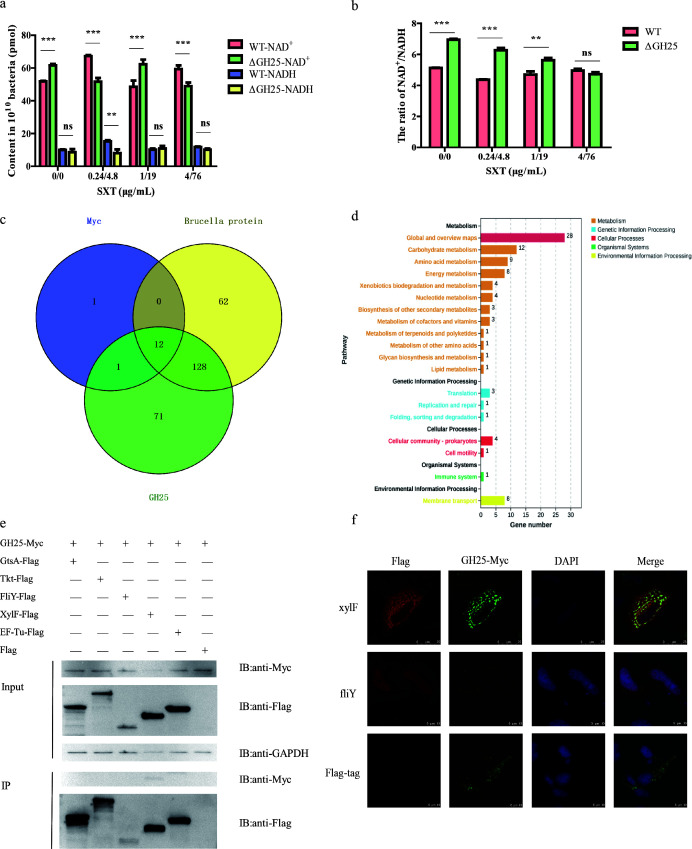
GH25-mediated SXT resistance of *B. melitensis* to SXT. After different concentrations of SXT treated to WT and ΔGH25 strains. (**a**) The content of NAD^+^ and NADH. (**b**) The ratio of NAD^+^/NADH. (**c**) The number of *B. melitensis* proteins screened and identified by pull-down and LC-MS/MS assays, and more than one peptide is selected for analysis. Myc is the amount of Myc-tag interacting with *Brucella* cultures, Brucella protein is the *Brucella* cultures interacting with magnetic beads, and GH25 is the amount of GH25 protein interacting with *Brucella* cultures (71 proteins). (**d**) The KEGG pathway functional classification of 71 proteins. (**e**) The Co-IP assay of the protein screened by multi-omics. (**f**) The result of laser confocal observation, and XylF colocalizes with GH25 protein. Significance levels: *P < 0.05, **P < 0.01, ***P < 0.001, and ns, not significant.

We next assessed the metabolic consequences of GH25 deletion (Fig. 5a and b; Fig. S3). After SXT exposure, the GH25-deletion strain showed significantly lower NADH levels and a higher NAD^+^/NADH ratio (Fig. 5a and b). This finding suggested impaired energy metabolism (26). To define the molecular mechanism of this metabolic change, we expressed tagged GH25 *in vitro* and identified GH25-interacting proteins via pull-down and LC-MS/MS analysis. Among 71 candidate proteins enriched in carbohydrate/amino acid metabolism and ABC transporters (Fig. 5c and d), we focused on proteins that could be linked to transport and energy use. This study identified sugar transport ABC transporter substrate-binding protein, XylF (DK62_RS15430), that was interacting with GH25 (Fig. 5e and f) by Co-IP and confocal laser scanning microscopy analysis. Transcriptomic analyses confirmed decreased XylF expression in resistant strains under SXT exposure (Table S3). Neural network predictions corroborated XylF as a potential SMZ target (Table S4). These findings suggest that GH25 may contribute to SXT resistance by altering energy metabolism through its interaction with XylF.

### GH25 mutation does not affect *B. melitensis* virulence

**Fig 6:**
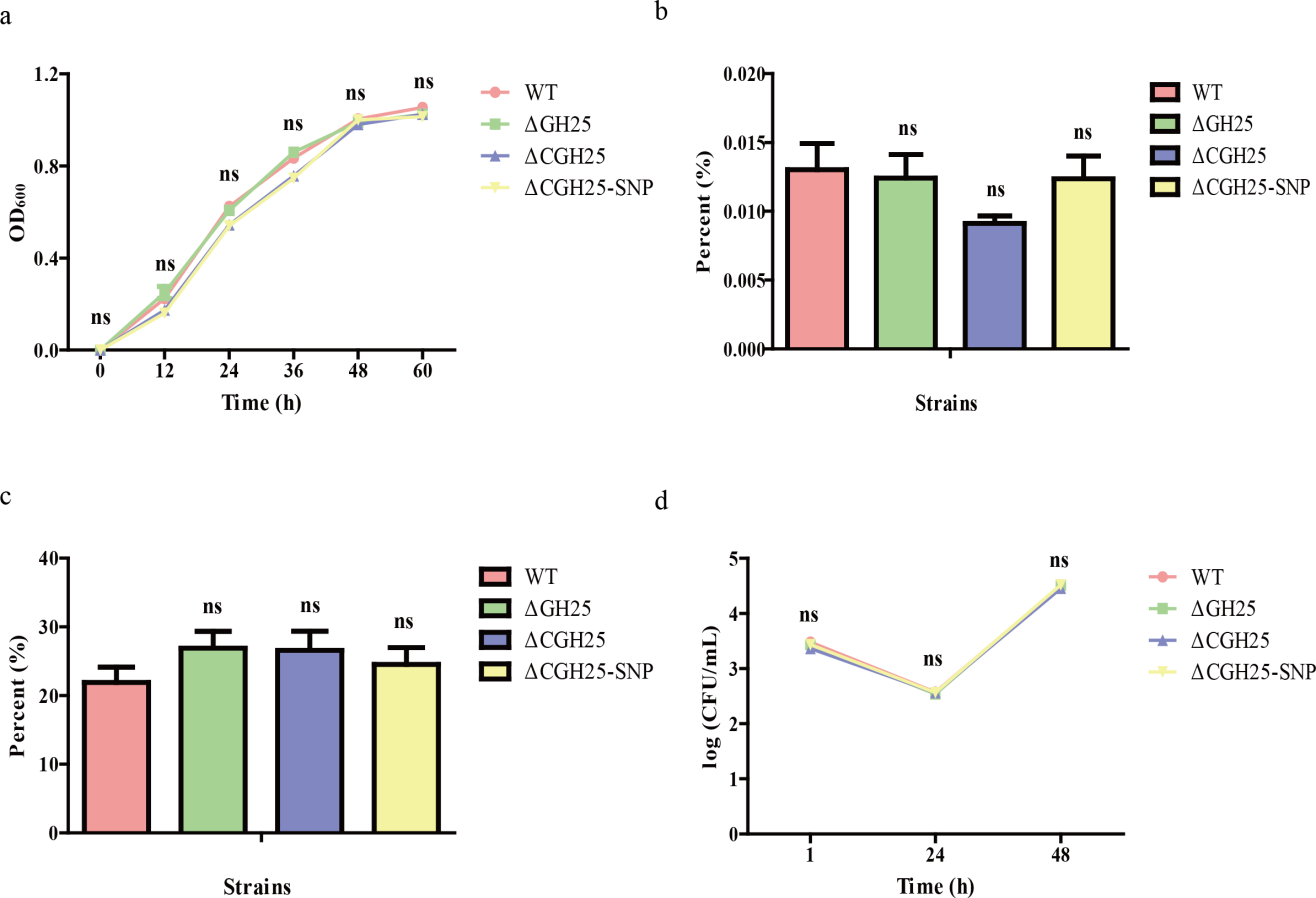
GH25 gene is not associated with *Brucella* virulence. (**a**) The growth curve of different strains. (**b**) The adhesion of different strains to RAW264.7 cells. (**c**) The invasion of different strains. (**d**) The intracellular survival of different strains in RAW264.7 cells at different time points. ns, not significant.

To determine whether GH25 protein affects virulence, the cell infection assay was performed among different constructed strains in RAW264.7 cells. No significant differences among the WT, GH25-deletion, GH25-complemented, and GH25-SNP-complemented strains in growth characteristics (Fig. 6a), adhesion (Fig. 6b), invasion (Fig. 6c), or intracellular survival (Fig. 6d) were observed. Thus, the GH25 mutation increases SXT resistance but does not reduce virulence.

### Mechanism of SXT resistance in Chinese *Brucella* isolates

**Fig 7:**
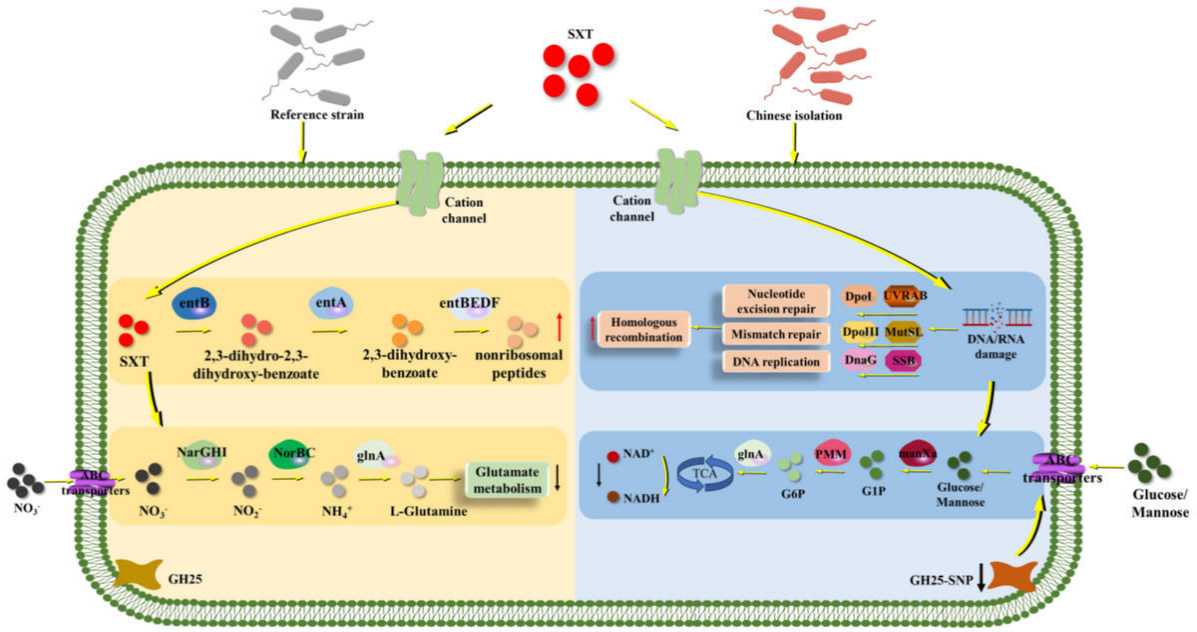
Proposed SXT resistance mechanism in Chinese *B. melitensis* isolates. Yellow arrows depict general pathways, red arrows indicate pathways with increased expression, and black arrows indicate pathways with decreased expression. Metabolic pathways in the reference strain (yellow box) and Chinese isolates (blue box) upon SXT exposure are highlighted. The blue box denotes the GH25 gene mutation and subsequent decreased expression, significantly impacting resistance mechanisms.

In the reference strain, SXT exposure induces siderophore nonribosomal peptides and suppresses nitrogen metabolism. In Chinese clinical isolates, long-term exposure to low levels of SXT selected for the GH25 frameshift mutations (A183del). This SNP truncated the C-terminal domain (beyond residue 218), altered interaction with the sugar-transport protein XylF, and rewired energy metabolism. At the same time, resistant isolates upregulated DNA repair, replication, and pyrimidine-metabolism pathways. These changes together reduced the sensitivity of the isolates to SXT (Fig. 7). Importantly, GH25 mutation did not impair infectivity, which may facilitate the spread of SXT-reduced-susceptibility strains.

## DISCUSSION

This study systematically evaluated the current status and mechanisms of SXT resistance in clinical *B. melitensis* isolates from China. We showed that SXT MICs have increased over time and that high-MIC isolates are spreading geographically. This resistance trend aligns with global observations, where nearly all *Brucella* isolates before 2000 remained sensitive to SXT (27, 28), but resistance reports have progressively increased since 2010 (29, 30). Clinical studies have shown that doxycycline with SXT can be as effective as doxycycline with rifampicin for non-focal brucellosis (31). However, in the Middle Eastern and North African countries, *Brucella* spp. remains *in vitro* susceptible to most antibiotics and combinations recommended by the WHO (32).

Previous reports on SXT-resistant strains in China (12, 13) have shown discrepancies due to differences in methodologies, quality control strains, and culture conditions. CLSI recommends broth microdilution (incubation at 35°C for 48 h with *E. coli* ATCC 25922 or *Streptococcus pneumoniae* ATCC 49619) as the standard for drug sensitivity testing in *Brucella* spp. Variations in testing methods (*E*-test or broth microdilution), quality control strains (e.g., *E. coli* ATCC 25922 and *Haemophilus influenzae* ATCC 10211I), and culture conditions (35°C or 37°C, ambient air or CO_2_ incubator) likely contribute to inconsistent resistance findings. For instance, resistance detected in Brucella broth medium contrasted with sensitivity detected using cation-adjusted Mueller-Hinton broth supplemented with lysed horse blood (33). Detection of Chinese *B. melitensis* isolates using Brucella broth microdilution found most of them sensitive to SXT (34). Globally, there are similar issues with SXT-resistant strains. Most resistance detection methods are *E*-test (35–37), or Kirby-Bauer disc diffusion method (Mueller-Hinton Agar) (38, 39), which are inconsistent. These discrepancies highlight the urgent need to standardize AST (antimicrobial susceptibility testing) for *Brucella*, including uniform media, incubation conditions, and annual QC-strain distribution. This study identified novel mechanisms underpinning SXT resistance. Known resistance mechanisms to SXT typically involve permeability barriers, efflux pumps, insensitive or modified target enzymes, and acquisition of resistant enzymes (40). This study specifically identified a mutation in the GH25 gene (183delA), resulting in a frameshift mutation from amino acid position 218 in resistant isolates. Bioinformatics analysis predicted that this protein has a lipoprotein signal peptide (Sec/SPII) (https://services.healthtech.dtu.dk/services/SignalP-5.0/), indicating that it may be secreted by *Brucella*. In addition, no transmembrane domain was predicted (https://services.healthtech.dtu.dk/services/TMHMM-2.0/), and the protein was predicted to localize to the bacterial inner membrane. (http://www.csbio.sjtu.edu.cn/bioinf/Gneg-multi/). GH25 encodes a lysozyme (EC 3.2.1.17) (41, 42), previously uncharacterized in *Brucella* spp. but extensively studied in other bacteria and fungi. In *Aspergillus fumigatus*, GH25 protein had a modified alpha/beta-barrel-like fold, and its active site lies toward the bottom of a negatively charged pocket (43). In *Mycobacterium tuberculosis*, GH25 is a Tat system-dependent secreted protein related to cell wall metabolism and sensitivity to β-lactams (44). In *Bacillus anthracis*, GH25 modulates the chemical alteration of peptidoglycan during bacterial cell growth and division (45). We therefore speculate that GH25 deletion or truncation may alter the cell-wall/peptidoglycan structure of *B. melitensis*. This structural change could make it harder for SXT to reach or accumulate at its target sites, indirectly increasing MICs. This hypothesis is supported by our LDH-release (Fig. S3) and energy-metabolism data.

Moreover, GH25 deletion impacted bacterial energy metabolism by significantly reducing NADH content, a factor previously linked to antibiotic resistance mechanisms (46). Interaction assays identified XylF, a sugar transport-related ABC system protein, as a GH25-interacting protein. This interaction indicates metabolic alterations, particularly in sugar transport and metabolism, may enhance antibiotic resistance. Similar metabolic adaptations influencing antibiotic resistance have been observed in *E. coli* (47), where specific carbon sources increased antibiotic resistance (48), and metabolic intermediates (e.g., malate and fumarate) influenced bacterial susceptibility (49).

Despite the complexity of resistance-associated mutations, single-gene deletion validation in reference strains confirmed GH25’s significant role. Further investigations into additional high-impact mutations (identified among the 79 genes potentially linked to resistance) are warranted. Additionally, our findings suggest the presence of more intricate resistance mechanisms, given the emergence of rough phenotype SXT-resistant *B. melitensis* isolates (2/38 μg/mL) not accounted for by GH25 mutations alone.

In conclusion, this study analyzing 636 *B. melitensis* isolates from various regions in China (2010–2021) revealed progressively increasing MICs to SXT. Specific SNPs were identified, enhancing nucleic acid repair and metabolic adaptations that confer resistance. GH25, influenced by specific SNPs, emerged as a critical mediator of resistance by interacting with XylF and impacting energy metabolism under SXT stress. Given the public health implications, rigorous surveillance and standardization of antibiotic resistance testing in *Brucella* spp. are imperative to prevent widespread resistance and protect public health globally.

## Data Availability

The transcriptome data of reference and Chinese isolated strains with/without SXT exposure have been deposited at the Sequence Read Archive (https://www.ncbi.nlm.nih.gov/sra/) under BioProject ID PRJNA1295374. The mass spectrometry proteomics data have been deposited to the ProteomeXchange Consortium (https://proteomecentral.proteomexchange.org) via the iProX (50) partner repository with the dataset identifier PXD066519. All of these data are available from the first author and corresponding authors upon reasonable request.

## SUPPLEMENTAL MATERIAL

Fig. S1 aac.01284-25-s0001.tif Functional annotation of common 179 SNPs.

Fig. S2 aac.01284-25-s0002.tif Construction and expression validation of GH25 deletion and complementation strains.

Fig. S3 aac.01284-25-s0003.tif Biochemical characterization of GH25 deletion and wild type strains.

Supplemental material aac.01284-25-s0004.docx Supplemental figure legends.

Supplemental tables aac.01284-25-s0005.xlsx Tables S1 to S4.

ASM does not own the copyrights to Supplemental Material that may be linked to, or accessed through, an article. The authors have granted ASM a non-exclusive, world-wide license to publish the Supplemental Material files. Please contact the corresponding author directly for reuse.

## References

[1] Nelson-Jones A. 1952. Brucellosis. Postgrad Med J 28:529–534. doi:10.1136/pgmj.28.324.529 13003643 PMC2530716

[2] Pappas G, Akritidis N, Bosilkovski M, Tsianos E. 2005. Brucellosis. N Engl J Med 352:2325–2336. doi:10.1056/NEJMra050570 15930423

[3] Laine CG, Johnson VE, Scott HM, Arenas-Gamboa AM. 2023. Global estimate of human brucellosis incidence. Emerg Infect Dis 29:1789–1797. doi:10.3201/eid2909.230052 37610167 PMC10461652

[4] Bukhari EE. 2018. Pediatric brucellosis. an update review for the new millennium. Saudi Med J 39:336–341. doi:10.15537/smj.2018.4.21896 29619483 PMC5938645

[5] Rohr JR, Barrett CB, Civitello DJ, Craft ME, Delius B, DeLeo GA, Hudson PJ, Jouanard N, Nguyen KH, Ostfeld RS, Remais JV, Riveau G, Sokolow SH, Tilman D. 2019. Emerging human infectious diseases and the links to global food production. Nat Sustain 2:445–456. doi:10.1038/s41893-019-0293-3 32219187 PMC7091874

[6] Jiang H, O’Callaghan D, Ding JB. 2020. Brucellosis in China: history, progress and challenge. Infect Dis Poverty 9:55. doi:10.1186/s40249-020-00673-8 32448394 PMC7247241

[7] Al-Tawfiq JA, Memish ZA. 2013. Pregnancy associated brucellosis. Recent Pat Antiinfect Drug Discov 8:47–50. doi:10.2174/1574891x11308010009 22812618

[8] Roushan MR, Amiri MJ. 2013. Update on childhood brucellosis. Recent Pat Antiinfect Drug Discov:42–46. doi:10.2174/1574891x11308010008 22812616

[9] Solera J, Martínez-Alfaro E, Espinosa A. 1997. Recognition and optimum treatment of brucellosis. Drugs (Abingdon Engl) 53:245–256. doi:10.2165/00003495-199753020-00005 9028744

[10] Sköld O. 2000. Sulfonamide resistance: mechanisms and trends. Drug Resist Updat 3:155–160. doi:10.1054/drup.2000.0146 11498380

[11] Qureshi KA, Parvez A, Fahmy NA, Abdel Hady BH, Kumar S, Ganguly A, Atiya A, Elhassan GO, Alfadly SO, Parkkila S, Aspatwar A. 2023. Brucellosis: epidemiology, pathogenesis, diagnosis and treatment–a comprehensive review. Ann Med 55. doi:10.1080/07853890.2023.2295398 PMC10769134 38165919

[12] Liu Z, Di D, Wang M, Liu R, Zhao H, Piao D, Zhao Z, Hao Y, Du Y, Jiang H, Cui B, Xia X. 2018. In vitro antimicrobial susceptibility testing of human Brucella melitensis isolates from Ulanqab of Inner Mongolia, China. BMC Infect Dis 18. doi:10.1186/s12879-018-2947-6 PMC5771123 29338693

[13] Yuan H-T, Wang C-L, Liu L-N, Wang D, Li D, Li Z-J, Liu Z-G. 2020. Epidemiologically characteristics of human brucellosis and antimicrobial susceptibility pattern of Brucella melitensis in Hinggan League of the Inner Mongolia Autonomous Region, China. Infect Dis Poverty 9. doi:10.1186/s40249-020-00697-0 PMC7325291 32600403

[14] Johansen TB, Scheffer L, Jensen VK, Bohlin J, Feruglio SL. 2018. Whole-genome sequencing and antimicrobial resistance in Brucella melitensis from a Norwegian perspective. Sci Rep 8:8538. doi:10.1038/s41598-018-26906-3 29867163 PMC5986768

[15] Yang X, Piao D, Mao L, Pang B, Zhao H, Tian G, Jiang H, Kan B. 2020. Whole-genome sequencing of rough Brucella melitensis in China provides insights into its genetic features. Emerg Microbes Infect 9:2147–2156. doi:10.1080/22221751.2020.1824549 32936049 PMC7580580

[16] Yang X, Wang N, Cao X, Bie P, Xing Z, Yin S, Jiang H, Wu Q. 2020. First isolation and characterization of Brucella suis from yak. Genome 63:397–405. doi:10.1139/gen-2019-0101 32384250

[17] Yang X, Wu T, Liu W, Tian G, Zhao H, Piao D, Jiang H, Wu Q. 2020. Cell membrane components of Brucella melitensis play important roles in the resistance of low-level rifampicin. PLoS Negl Trop Dis 14:e0008888. doi:10.1371/journal.pntd.0008888 33373362 PMC7771680

[18] Huang H, Zhang G, Zhou Y, Lin C, Chen S, Lin Y, Mai S, Huang Z. 2018. Reverse screening methods to search for the protein targets of chemopreventive compounds. Front Chem 6. doi:10.3389/fchem.2018.00138 PMC5954125 29868550

[19] Yuan Y, Ning W, Chen J, Li J, Xue T, An C, Mao L, Zhang G, Zhou S, Ding J, Yang X, Ye J. 2025. Serine/threonine protein kinase mediates rifampicin resistance in Brucella melitensis through interacting with ribosomal protein RpsD and affecting antioxidant capacity. mSystems 10. doi:10.1128/msystems.01109-24 PMC11748488 39636113

[20] Yang X, Wang J, Feng Z, Zhang X, Wang X, Wu Q. 2019. Relation of the pdxB-usg-truA-dedA operon and the truA gene to the intracellular survival of Salmonella enterica serovar typhimurium. IJMS 20:380. doi:10.3390/ijms20020380 30658401 PMC6358828

[21] Hindler JF, Stelling J. 2007. Analysis and presentation of cumulative antibiograms: a new consensus guideline from the Clinical and Laboratory Standards Institute. Clin Infect Dis 44:867–873. doi:10.1086/511864 17304462

[22] Biswas S, Raoult D, Rolain J-M. 2008. A bioinformatic approach to understanding antibiotic resistance in intracellular bacteria through whole genome analysis. Int J Antimicrob Agents 32:207–220. doi:10.1016/j.ijantimicag.2008.03.017 18619818

[23] Poey ME, de Los Santos E, Aznarez D, García-Laviña CX, Laviña M. 2024. Genetics of resistance to trimethoprim in cotrimoxazole resistant uropathogenic Escherichia coli: integrons, transposons, and single gene cassettes. Front Microbiol 15:1395953. doi:10.3389/fmicb.2024.1395953 38946902 PMC11213556

[24] Yang X, Wang Y, Li J, Chen J, Liu J, Tian G, Zhao H, Piao D, Fan Y, Jiang H. 2022. Genetic characteristics of an amikacin-resistant Brucella abortus strain first isolated from Marmota himalayana. Microb Pathog 164:105402. doi:10.1016/j.micpath.2022.105402 35038548

[25] Hall MB, Rabodoarivelo MS, Koch A, Dippenaar A, George S, Grobbelaar M, Warren R, Walker TM, Cox H, Gagneux S, Crook D, Peto T, Rakotosamimanana N, Grandjean Lapierre S, Iqbal Z. 2023. Evaluation of nanopore sequencing for Mycobacterium tuberculosis drug susceptibility testing and outbreak investigation: a genomic analysis. The Lancet Microbe 4:e84–e92. doi:10.1016/S2666-5247(22)00301-9 36549315 PMC9892011

[26] Zhu S, Yang B, Yu F, Zhang J, Wang Z, Liu Y. 2024. Investigation of the impact of widely used pesticides on conjugative transfer of multidrug resistance plasmids. J Hazard Mater 478:135436. doi:10.1016/j.jhazmat.2024.135436 39141944

[27] Qadri H, Halim MA, Ueno Y, Abumustafa FM, Postle AG. 1995. Antibacterial ctivity of azithromycin against Brucella melitensis. Chemotherapy 41:253–256. doi:10.1159/000239353 7555205

[28] Hall WH. 1990. Modern chemotherapy for brucellosis in humans. Rev Infect Dis 12:1060–1099. doi:10.1093/clinids/12.6.1060 2267485

[29] Cama BAV, Ceccarelli M, Venanzi Rullo E, Ferraiolo F, Paolucci IA, Maranto D, Mondello P, Lo Presti Costantino MR, Marano F, D’Andrea G, Di Marco V, Puglisi G, Valenzise M, D’Angelo G, Mondello L, Strano G, Condorelli F, Spicola D, Nunnari G, Pellicanò GF. 2019. Outbreak of Brucella melitensis infection in Eastern Sicily: risk factors, clinical characteristics and complication rate. New Microbiol 42:43–48. doi:10.13140/RG.2.2.22561.66404 30957869

[30] Celik E, Kayman T, Buyuk F, Gulmez Saglam A, Abay S, Akar M, Karakaya E, Balkan Bozlak CE, Coskun MR, Buyuk E, Celebi O, Sahin M, Saticioglu IB, Durhan S, Baykal A, Ersoy Y, Otlu S, Aydin F. 2023. The canonical Brucella species-host dependency is changing, however, the antibiotic susceptibility profiles remain unchanged. Microb Pathog 182:106261. doi:10.1016/j.micpath.2023.106261 37488036

[31] Edathodu J, Alamri M, Alshangiti KA, Alfagyh NS, Alnaghmush AS, Albaiz F, Alothman B, Khalil H, Edathodu Z, Alrajhi AA. 2021. Clinical manifestations and treatment outcomes of human brucellosis at a tertiary care center in Saudi Arabia. Ann Saudi Med 41:109–114. doi:10.5144/0256-4947.2021.109 33818142 PMC8020648

[32] Wareth G, Dadar M, Ali H, Hamdy MER, Al‐Talhy AM, Elkharsawi AR, Tawab AAAE, Neubauer H. 2022. The perspective of antibiotic therapeutic challenges of brucellosis in the Middle East and North African countries: current situation and therapeutic management. Transbounding Emerging Dis 69. doi:10.1111/tbed.14502 35244335

[33] Arapović J, Kompes G, Dedić K, Teskeredžić S, Ostojić M, Travar M, Tihić N, Delić J, Skočibušić S, Zekiri-Sivro M, Verhaz A, Piljić D, Laura L, Duvnjak S, Zdelar-Tuk M, Arapović M, Šabotić E, Reil I, Nikolić J, Ahmetagić S, Cvetnić Ž, Habrun B, Bosilkovski M, Špičić S. 2022. Antimicrobial resistance profiles of human Brucella melitensis isolates in three different microdilution broths: the first multicentre study in Bosnia and Herzegovina. J Glob Antimicrob Resist 29:99–104. doi:10.1016/j.jgar.2022.02.005 35182775

[34] Jiang H, Mao L, Zhao H, Li L, Piao D, Yao W, Cui B. 2010. MLVA typing and antibiotic susceptibility of Brucella human isolates from Liaoning, China. Trans R Soc Trop Med Hyg 104:796–800. doi:10.1016/j.trstmh.2010.08.002 20832094

[35] Maves RC, Castillo R, Guillen A, Espinosa B, Meza R, Espinoza N, Núñez G, Sánchez L, Chacaltana J, Cepeda D, González S, Hall ER. 2011. Antimicrobial susceptibility of Brucella melitensis isolates in Peru. Antimicrob Agents Chemother 55:1279–1281. doi:10.1128/AAC.00979-10 21199926 PMC3067062

[36] Köse Ş, Kiliç S, Özbel Y. 2005. Identification of Brucella species isolated from proven brucellosis patients in Izmir, Turkey. J Basic Microbiol 45:323–327. doi:10.1002/jobm.200410469 16028204

[37] Turkmani A, Ioannidis A, Christidou A, Psaroulaki A, Loukaides F, Tselentis Y. 2006. In vitro susceptibilities of Brucella melitensis isolates to eleven antibiotics. Ann Clin Microbiol Antimicrob 5. doi:10.1186/1476-0711-5-24 PMC1617115 17014707

[38] Ilhan Z, Solmaz H, Ekin IH. 2013. In vitro antimicrobial susceptibility of Brucella melitensis isolates from sheep in an area endemic for human brucellosis in Turkey. J Vet Med Sci 75:1035–1040. doi:10.1292/jvms.12-0163 23545462

[39] Barbosa Pauletti R, Reinato Stynen AP, Pinto da Silva Mol J, Seles Dorneles EM, Alves TM, de Sousa Moura Souto M, Minharro S, Heinemann MB, Lage AP. 2015. Reduced susceptibility to rifampicin and resistance to multiple antimicrobial agents among Brucella abortus Isolates from Cattle in Brazil. PLoS One 10:e0132532. doi:10.1371/journal.pone.0132532 26181775 PMC4504493

[40] Huovinen P. 2001. Resistance to trimethoprim-sulfamethoxazole. Clin Infect Dis 32:1608–1614. doi:10.1086/320532 11340533

[41] Henrissat B. 1991. A classification of glycosyl hydrolases based on amino acid sequence similarities. Biochem J 280 ( Pt 2):309–316. doi:10.1042/bj2800309 1747104 PMC1130547

[42] Henrissat B, Bairoch A. 1996. Updating the sequence-based classification of glycosyl hydrolases. Biochem J 316 (Pt 2):695–696. doi:10.1042/bj3160695 8687420 PMC1217404

[43] Korczynska JE, Danielsen S, Schagerlöf U, Turkenburg JP, Davies GJ, Wilson KS, Taylor EJ. 2010. The structure of a family GH25 lysozyme from Aspergillus fumigatus. Acta Crystallogr Sect F Struct Biol Cryst Commun 66:973–977. doi:10.1107/S1744309110025601 PMC2935209 20823508

[44] Bellinzoni M, Haouz A, Miras I, Magnet S, André-Leroux G, Mukherjee R, Shepard W, Cole ST, Alzari PM. 2014. Structural studies suggest a peptidoglycan hydrolase function for the Mycobacterium tuberculosis Tat-secreted protein Rv2525c. J Struct Biol 188:156–164. doi:10.1016/j.jsb.2014.09.003 25260828

[45] Martinez-Fleites C, Korczynska JE, Davies GJ, Cope MJ, Turkenburg JP, Taylor EJ. 2009. The crystal structure of a family GH25 lysozyme from Bacillus anthracis implies a neighboring-group catalytic mechanism with retention of anomeric configuration. Carbohydr Res 344:1753–1757. doi:10.1016/j.carres.2009.06.001 19595298

[46] Su Y, Peng B, Li H, Cheng Z, Zhang T, Zhu J, Li D, Li M, Ye J, Du C, Zhang S, Zhao X, Yang M, Peng X. 2018. Pyruvate cycle increases aminoglycoside efficacy and provides respiratory energy in bacteria. Proc Natl Acad Sci USA 115. doi:10.1073/pnas.1714645115 PMC5816162 29382755

[47] Allison KR, Brynildsen MP, Collins JJ. 2011. Metabolite-enabled eradication of bacterial persisters by aminoglycosides. Nature 473:216–220. doi:10.1038/nature10069 21562562 PMC3145328

[48] Amato SM, Orman MA, Brynildsen MP. 2013. Metabolic control of persister formation in Escherichia coli. Mol Cell 50:475–487. doi:10.1016/j.molcel.2013.04.002 23665232

[49] Meylan S, Porter CBM, Yang JH, Belenky P, Gutierrez A, Lobritz MA, Park J, Kim SH, Moskowitz SM, Collins JJ. 2017. Carbon sources tune antibiotic susceptibility in Pseudomonas aeruginosa via tricarboxylic acid cycle control. Cell Chem Biol 24:195–206. doi:10.1016/j.chembiol.2016.12.015 28111098 PMC5426816

[50] Chen T, Ma J, Liu Y, Chen Z, Xiao N, Lu Y, Fu Y, Yang C, Li M, Wu S, Wang X, Li D, He F, Hermjakob H, Zhu Y. 2022. iProX in 2021: connecting proteomics data sharing with big data. Nucleic Acids Res 50:D1522–D1527. doi:10.1093/nar/gkab1081 34871441 PMC8728291

