# Supplementary figures and images for: Novel metabolic adaptation driven by glycoside hydrolase family 25 protein contributes to increasing trimethoprim-sulfamethoxazole resistance in clinical human Brucella melitensis isolates in China
Source: Antimicrob Agents Chemother. 2026 Jan 22;70(3):e01284-25. doi: 10.1128/aac.01284-25 (PMC12959145; doi:10.1128/aac.01284-25)

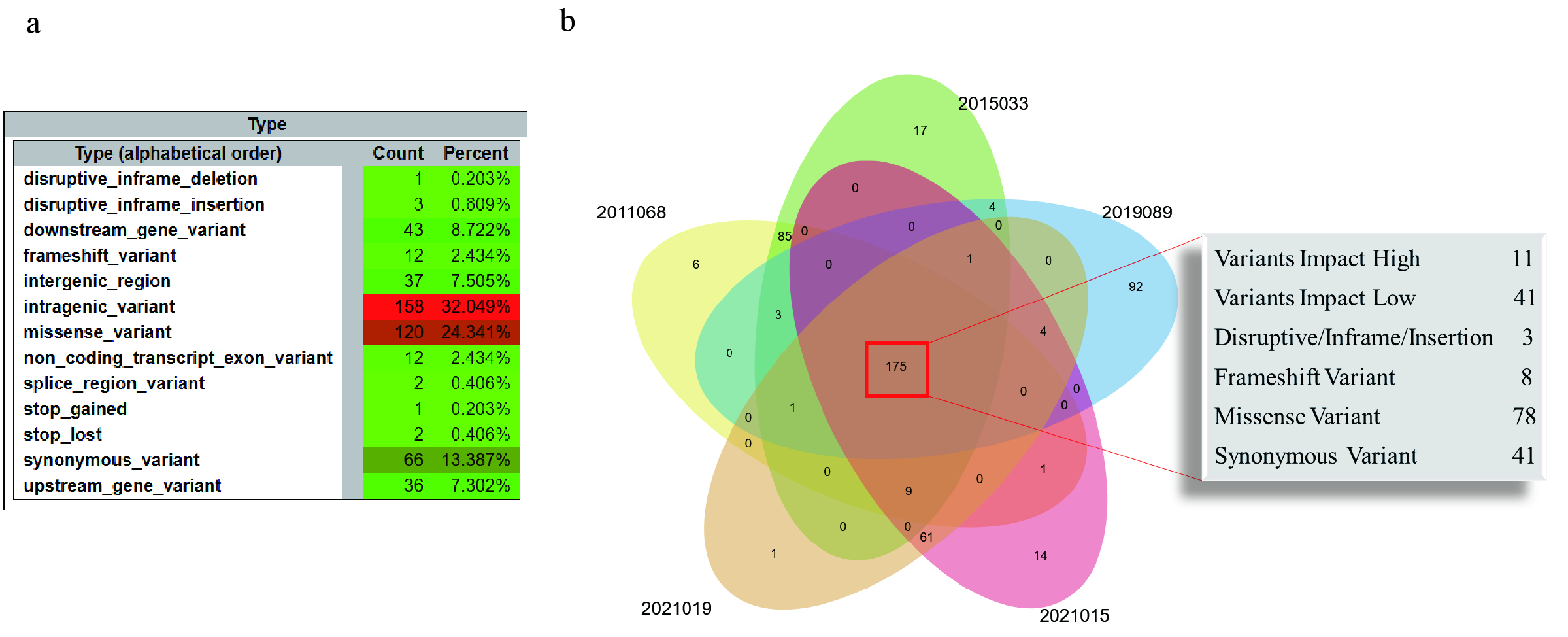

Supplement: Fig. S1 — Functional annotation of common 179 SNPs. [file aac.01284-25-s0001.tif]

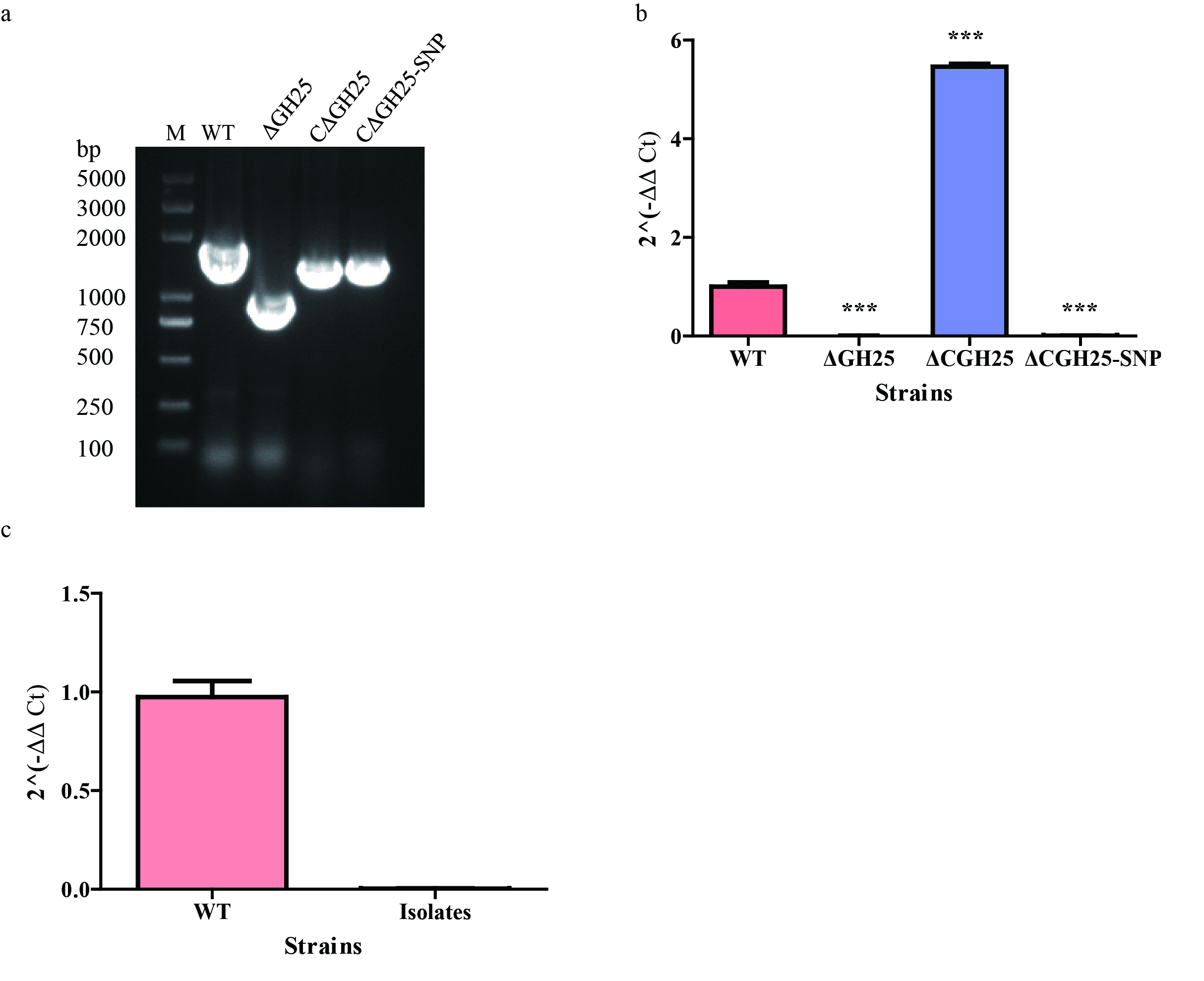

Supplement: Fig. S2 — Construction and expression validation of GH25 deletion and complementation strains. [file aac.01284-25-s0002.tif]

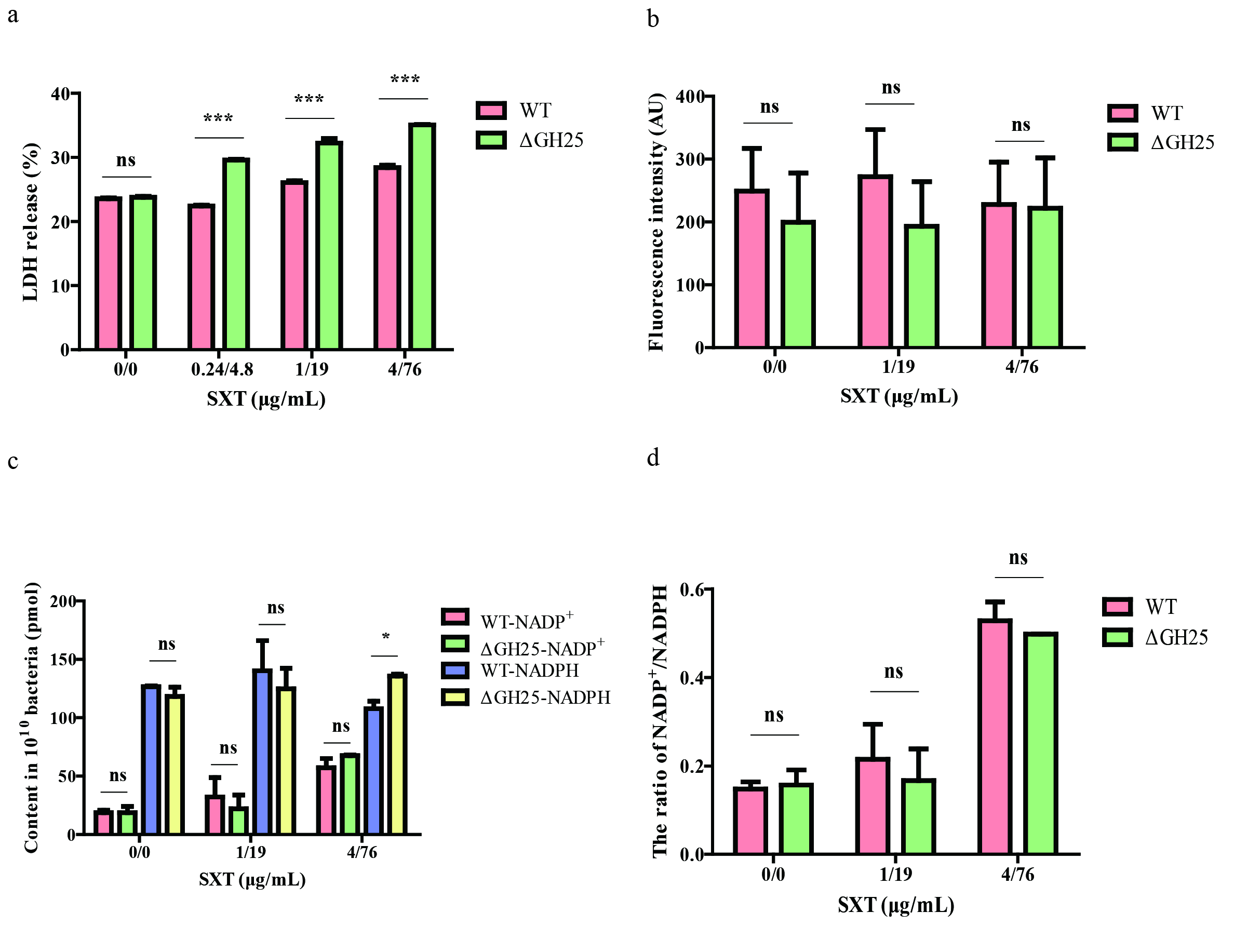

Supplement: Fig. S3 — Biochemical characterization of GH25 deletion and wild type strains. [file aac.01284-25-s0003.tif]
