# Supplementary material for: Novel metabolic adaptation driven by glycoside hydrolase family 25 protein contributes to increasing trimethoprim-sulfamethoxazole resistance in clinical human Brucella melitensis isolates in China
Source: Antimicrob Agents Chemother. 2026 Jan 22;70(3):e01284-25. doi: 10.1128/aac.01284-25 (PMC12959145; doi:10.1128/aac.01284-25)
Supplement: Supplemental material — Supplemental figure legends. [file aac.01284-25-s0004.docx]

**Figure S1 Functional annotation of common 179 SNPs.** (a) Annotation results of SNPs and InDels among Chinese isolates. (b) Analysis of SNP sites shared by five resistant isolates, categorizing the 175 SNP-affected genes.

**Figure S2 Construction and expression validation of GH25 deletion and complementation strains.** (a) PCR verification of successful construction of deletion, complementation, and mutant complementation strains. Primers upstream and downstream of the GH25 gene were used for WT and ΔGH25, while universal primers for pBBR1mcs plasmid were used for ΔCGH25 and ΔCGH25-SNP strains. Primer sequences are listed in Table S1. (b) and (c) Relative expression levels of GH25 in different strains. Total RNA was extracted from the strains, and the relative expression levels of GH25 in different strains were determined with the wild-type strain as the reference. (b) is the results of ΔGH25, ΔCGH25 and ΔCGH25-SNP strains, and (c) is the results of Chinese isolates.

**Figure S3 Biochemical characterization of GH25 deletion and wild type strains.** After treating WT and ΔGH25 strains with different concentrations of SXT, biochemical indicators were measured: (a) LDH release, (b) ROS content, (c) NADPH and NADP^+^ content, and (d) NADP^+^/NADPH ratios. Significance levels: * *p* < 0.05, ** *p* < 0.01, *** *p* < 0.001.
